# Vegetation loss and the 2016 Oropouche fever outbreak in Peru

**DOI:** 10.1590/0074-02760160415

**Published:** 2017-04

**Authors:** Daniel Romero-Alvarez, Luis E Escobar

**Affiliations:** 1 Hospital General Enrique Garcés, Unidad de Epidemiología, Quito, Ecuador; 2 University of Minnesota, Department of Fisheries, Wildlife and Conservation Biology, St. Paul, MN, USA

**Keywords:** Oropouche, ecological niche modeling, vegetation, outbreak, Culicoides, MODIS

## Abstract

**BACKGROUND:**

*Oropouche virus* causes Oropouche fever, an arboviral disease transmitted mainly by midges of the genus *Culicoides* and *Culex* mosquitoes. Clinical presentation of Oropouche fever in humans includes fever, headache, rash, myalgia, and in rare cases spontaneous bleeding and aseptic meningitis. Landscape change has been proposed as a driver of Oropouche fever emergence.

**OBJECTIVE:**

To investigate the landscape epidemiology of the Oropouche fever outbreak that began in April 2016 in Cusco, Peru.

**METHODS:**

We used information of vegetation and multivariate spatial analyses including ecological niche modeling. Vegetation was characterised using16-day composite enhanced vegetation index (EVI) images at 500 m spatial resolution from the MODIS sensor carried by the Terra satellite.

**FINDINGS:**

Cases were distributed across seven Peruvian districts in two provinces. La Concepcion was the province with most of the affected districts. EVI time series across 2000 to 2016 suggested a decline in the vegetation in sites with Oropouche fever cases before the epidemic. Our ecological niche modeling suggests that other areas in Junin, Apurimac, and Madre de Dios departments are at risk of Oropouche fever occurrence.

**MAIN CONCLUSIONS:**

Our results may provide a guide for future fieldwork to test hypotheses regarding Oropouche fever emergence and habitat loss in tropical Latin America.

*Oropouche virus* belongs to the family Bunyaviridae, genus *Orthobunyavirus*. At least four genotypes have been recognised for Oropouche in outbreaks in Trinidad and Tobago, Panama, Brazil and Peru (Vasconcelos et al. 2011). The virus produces Oropouche fever, an arboviral disease transmitted chiefly by the midge *Culicoides paraensis* in an urban transmission cycle (Pinheiro et al. 1981, 1982). The mosquito *Culex quinquefasciatus* has been also implicated as a human vector of Oropouche fever, but with limited competence (Cardoso et al. 2015). The sylvatic cycle of the disease includes long term circulation in sloths, rodents, monkeys and birds (Pinheiro et al. 1962, 1976, Nunes et al. 2005), with transmission by the mosquitoes *Cx. quinquefasciatus*, *Aedes serratus* and *Coquillettidia venezuelensis* as vectors among these wild animals (Anderson et al. 1961, Hoch et al. 1987).

Clinical presentation of Oropouche fever in humans includes fever, headache, myalgia, rash, spontaneous bleeding (in rare cases: petechias, epistaxis, and gingival bleeding) (Alvarez-Falconi & Ruiz 2010) and aseptic meningitis (Bastos et al. 2012). Oropouche fever should therefore be considered in differential diagnosis of South American febrile diseases like the endemic malaria, dengue, and yellow fever, and novel diseases like chikungunya and zika fevers. Although fatal cases have not been reported, Oropouche fever is known for causing dramatic human epidemics (Pinheiro et al. 1962, Alvarez-Falconi & Ruiz 2010). Peru reported Oropouche fever for the first time in 1992, and outbreaks have been reported in diverse Peruvian departments including San Martin, Cajamarca, Cusco and Madre de Dios (Alvarez-Falconi & Ruiz 2010, Castro et al. 2013, García et al. 2016, MINSA 2016). No ecological studies have been developed to date to understand factors facilitating these outbreaks.

Landscape change has been proposed as driver of emergence of diseases. Exploring land cover conditions under which infectious diseases occur may inform prevention strategies of disease emergence. To explore plausible factors facilitating Oropouche fever emergence, we developed an ecological survey of the Oropouche fever outbreak that began in April 2016 in Cusco, Peru. Our aims were (i) to explore possible landscape drivers that could explain the emergence event (descriptive analysis), and (ii) to identify other suitable areas for potential Oropouche fever transmission based on landscape conditions in the surroundings (predictive analysis). We collected geographic information on the outbreak, and assessed landscape characteristics using land cover information from remote sensing data and ecological niche modeling; results of these analyses can support efforts to contain the ongoing epidemic and anticipate future outbreaks for their prevention.

## MATERIALS AND METHODS

*Occurrences* - Case-occurrence records from the Cusco outbreak have been made available by the electronic reporting system of infectious diseases, ProMED (Madoff 2004). We searched in ProMED for the keywords ‘Oropouche,’ ‘Oropuche,’ and ‘Oropouche fever,’ from January to May 2016 (http://www.promedmail.org/). We included reports with geographic information regarding outbreak localities, which in turn were georeferenced using Google Earth (http://www.google.com/earth/).

*Study area* - The study area extent impacts correlative modeling outputs, making predictions area dependent (Barve et al. 2011). We selected a study area including all Oropouche fever outbreak localities with cases reported by May 2016 comprising a region between 74.99º W, 11.08º S and 70.89º W, 14.03º S. In this area, we explored landscape variations at outbreak sites to identify other areas potentially suitable for emergence of future Oropouche fever cases. To explore patterns of landscape characteristics in the study area, we used 16-day composite enhanced vegetation index (EVI) images at 500 m spatial resolution from the MODIS sensor (MOD13A1) on the Terra satellite (https://lpdaac.usgs.gov/data_access/data_pool). EVI captures land surface biophysical patterns and processes, including primary production and land cover change and provides consistent information on temporal and spatial patterns of vegetation features. EVI values range from -2,000 (low vegetation mass) to 10,000 (high vegetation mass).

*Descriptive analysis* - First, we explored spatial patterns of vegetation change based on two EVI layers: one of April 2015 and another of April 2016, as the latter is the month of the Oropouche epidemic in Cusco - Peru. The analysis of spatial changes in vegetation was developed via a principal component analysis (PCA) of these two EVI layers using the *Spatial Analysis Tool* in ArcGIS 10.3 (ESRI Redlands, CA) to summarise variation between both years in the same site and month. According to Horning et al. (2010), when using a vegetation index time series as we do here, the second principal component from a PCA transformation provides information on changes in land cover over time. While this method allows one to identify small changes in vegetation composition, visual interpretation of changes is necessary to detect deforestation or reforestation in the pixels of the second component (Horning et al. 2010). The use of PCA for land cover classification has been the most accurate method with above 90% overall accuracy for identifying vegetation type and change; and is better than unsupervised and supervised classification methods (Horning et al. 2010). Secondly, we used a Student’s *t-*test to quantitatively explore the difference between these layers for Oropouche fever infected localities vs. non-infected localities (see below) with *α* = 0.05.

Finally, we explored temporal patterns of vegetation change using time series from 2000 to 2016 of EVI data for the month of April. Using these data, we developed a ~2 km buffer area around locations of Oropouche fever cases, as this distance approximates estimates of the dispersal capacity of *Cs. paraensis* (Elbers et al. 2015). We generated 100 random points in these areas and estimated the total and average EVI values across epidemic sites. To account for general landscape features across the study area, we also selected nine localities without Oropouche fever cases reported at the time this study was developed.

*Predictive analyses* - To forecast areas with potential for Oropouche fever outbreaks we employed an ecological niche modeling approach. Ecological niche models have been applied successfully to forecast infectious disease transmission risk (e.g., Peterson & Samy 2016). Briefly, ecological niche models correlate disease occurrence reports with environmental information in the study area to recognise suitable areas similar to those where the disease was reported. We developed the ecological niche model using Maxent 3.3.3k. Maxent identifies suitable areas based on the probability distribution of maximum entropy, subject to environmental constraints (Phillips & Dudík 2008). For model calibration, we used locations of Oropouche fever cases and EVI data as environmental constraints. For this analysis, we used bi-weekly EVI layers between January 2015-April 2016 to summarise landscape conditions for 15 months prior the epidemic. To reduce both the dimensionality of and correlations among EVI layers, we developed a PCA from the original 30 bi-weekly EVI layers using NicheA software version 3.0 (http://nichea.sourceforge.net/whatsnew.html), and used the first four principal components summarising > 80% of overall variance as environmental data layers in the niche model development.

The ecological niche model was calibrated via 25 bootstrap replicates in Maxent. Each replicate was based on 80% of occurrences, reserving 20% for internal evaluations. As final predictions, we selected the median of replicates in logistic format in Maxent, and used this value as a suitability index for Oropouche occurrence. Because Maxent is prone to overfitting under default parameters (Radosavljevic & Anderson 2014), we tuned its regularisation coefficients to identify the best model fit with reduced parametrisation. We assessed the regularisation coefficients 0.5, 1, 1.5, and 2, and selected that with the lowest Akaike Information Criterion value corrected by the number of samples (AICc). This value identifies the regularisation coefficient that provides the appropriate level of complexity and best fit to the available data, considering the minimum possible lost of information. AICc values were calculated in ENMTools (http://enmtools.blogspot.com/) as described elsewhere (Warren & Seifert 2011).

## RESULTS

**Fig. 1:**
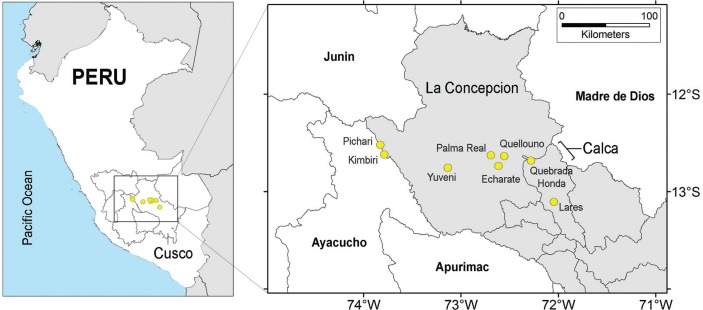
: location of the Oropouche fever epidemic in Peru, 2016. Oropouche fever occurrences shown as yellow points; study area extent indicated by the rectangle. Affected communities Pichari, Kimbiri, Yuveni, Palma Real, Echarate, and Quellouno occur in La Concepcion province; Quebrada Honda and Lares occur in Calca province. Both provinces belong to Cusco department (grey shading in the right).

**Fig. 2:**
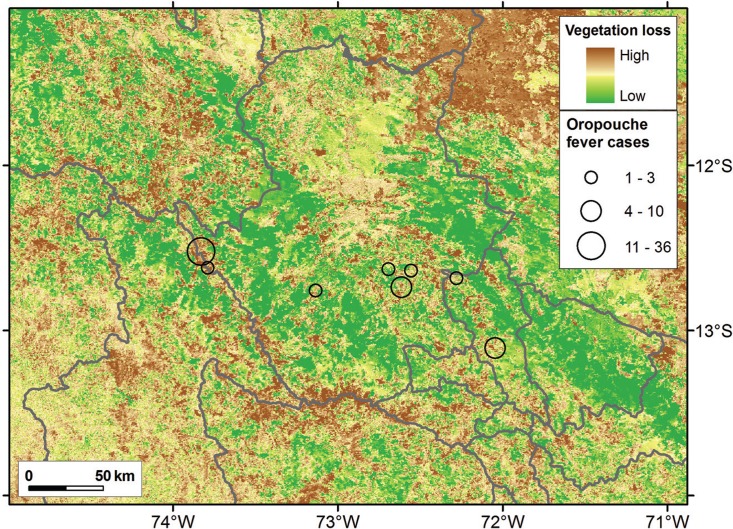
: vegetation status in Oropouche fever positive localities. High loss (brown), stability (yellow), and gain (green) of vegetation determined by the second component from a principal component analysis of enhanced vegetation index (EVI) April 2015 and April 2016. Oropouche fever cases in each outbreak location are insert (black circles). Locations are the same as in Fig. 1.

ProMED reported 61 cases of Oropouche (Table I). Cases were distributed across seven Peruvian districts in two provinces: La Concepcion and Calca, within Cusco department (Fig. 1). Vegetation loss was higher in outbreak locations compared with free-case localities in terms of vegetation differences between April 2015 and April 2016 (*t* = -4.97, df = 1652.4, p < 0.001), particularly in the area around Pichari and Kimbiri (Fig. 2). Pichari was the urban settlement where the majority of cases were reported to date (Table I). La Concepcion was the province with most affected districts; indeed, six of the seven outbreak sites occurred in La Concepcion (Table I).

**TABLE I:** Location of Oropouche fever outbreaks in 2016

Province	District	Urban settlement	Cases	Longitude	Latitude
La Concepcion	Pichari	Pichari	36	-73.827843	-12.520314
	Kimbiri	Kimbiri	2	-73.787498	-12.618733
	Yuveni	Yuveni	3	-73.134699	-12.75572
	Quellouno	Quellouno	1	-72.555899	-12.635893
	Echarate	Palma Real	1	-72.69288	-12.626443
		Echarate	6	-72.614288	-12.735741
Calca	Lares	Lares	10	-72.044594	-13.105302
	Yanalite	Quebrada Honda	2	-72.279309	-12.681124

**Fig. 3:**
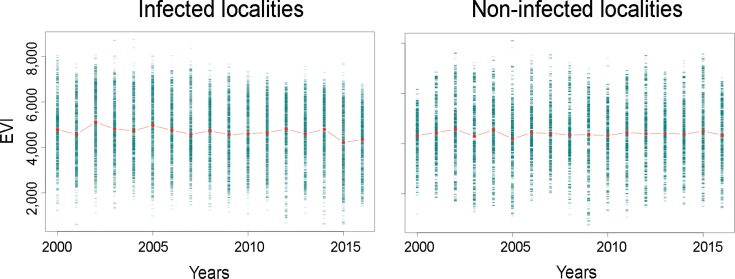
: vegetation composition in sites with and without Oropouche fever outbreaks 2000-2016. Vegetation status from 2000 to 2016 considering a ~2 km buffer around the localities of the current Oropouche fever epidemic and nine locations without cases. Enhanced vegetation index (EVI) values by April of each year are displayed in raw (green) and mean values (red points). Notice the loss of vegetation a year before 2015 in positive localities (left panel).

**Fig. 4:**
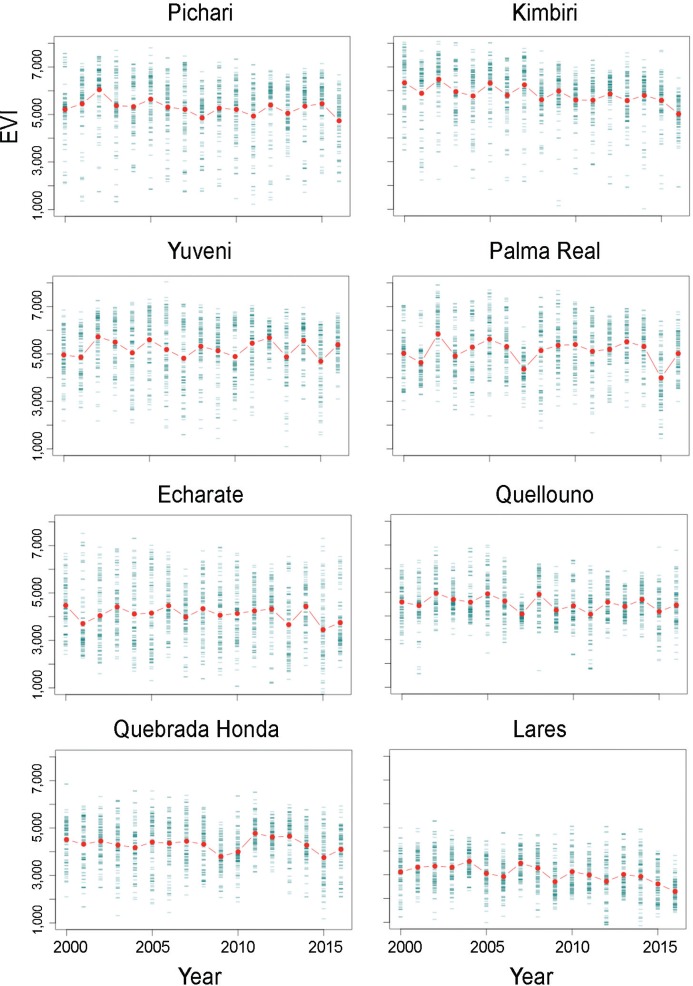
: landscape variation by location reporting Oropouche fever cases. Plots represent enhanced vegetation index (EVI) values in the positive localities during April between 2000-2016. A considerable reduction is noticeable in all locations by 2015 or 2016.

EVI time series exploration showed a decline of vegetation in sites with Oropouche fever cases before the epidemic. We notice a small decline of vegetation by 2016 (Fig. 3). Vegetation reduction was clear in Pichari, Kimbiri, Echarate, and Lares (Fig. 4); Lares was the second location in number of cases (Table I). All localities showed vegetation loss since 2015; Palma Real showed a noticeable loss by 2015 and an apparent increment by 2016.

**Fig. 5:**
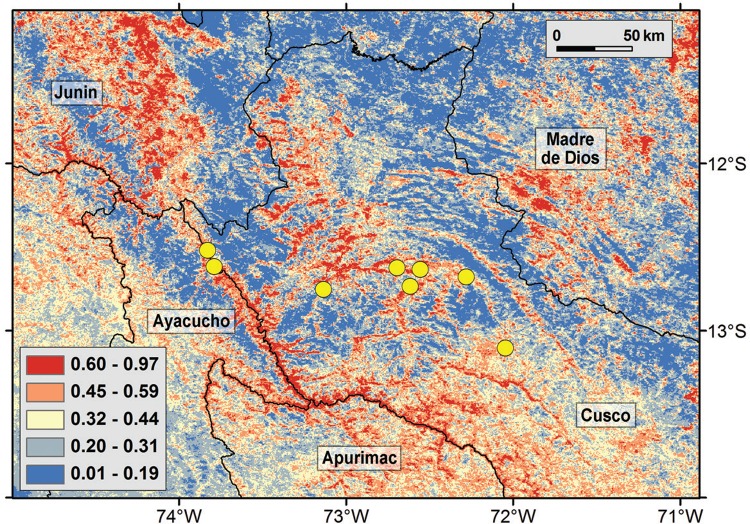
: ecological niche model of Oropouche fever cases. Areas with high (red) and low suitability (blue) were identified based on enhanced vegetation index (EVI) values. High suitability was observed across the border between Cusco-Apurimac-Ayacucho (i.e., Apurimac river), and in areas of the northwest (Junin), northeast (Madre de Dios), and south (Apurimac) regions.

The ecological niche model required a regularisation coefficient of 0.5 for the best fit (Table II). The final model indicated that the northwestern parts of the study area have high suitability for Oropouche fever transmission, specifically in Junin department, and also in the southern part of the study area in Apurimac department (Fig. 5). Areas highly suitable were also found in the northeastern parts of Madre de Dios department.

**TABLE II:** Akaike information criterion (AICc) values corrected by sample size. In bold the lowest AICc value that identifies the best regularisation coefficient for the development of the final model

Regularisation coefficients	Parameters	Sample size	AICc
0.5	3	8	**218.60**
1	3	8	220.16
1.5	2	8	218.65
2	2	8	218.87

## DISCUSSION

We present a first eco-epidemiological assessment of the recent outbreak of Oropouche fever in Cusco, Peru. Oropouche-positive localities tended to have experienced vegetation loss before the beginning of the outbreak in comparison with case-free sites. Ecological niche models, however, identified sites presenting suitable conditions for Oropouche fever transmission in the neighboring departments of Junin and Apurimac, suggesting risk for further disease translocation into nearby unaffected but suitable areas.

We found limited information regarding Oropouche fever vectors in Peru. To the best of our knowledge, midges, including *Cs. paraensis*, have not been found infected with Oropouche virus in the study area. For instance, several research teams have conducted entomological surveys around Iquitos in Loreto department, without finding the virus in the midges captured (Turell et al. 2005). Moreover, records of Oropouche fever cases in the neighboring department of Madre de Dios also lack information about the vector species implicated in the outbreaks (García et al. 2016). On the other hand, Oropouche fever research in Brazil has found *Cs. paraensis* Oropouche virus positive in outbreak localities (Pinheiro et al. 1976, Vasconcelos et al. 1989); however, a considerable number of midges were necessary for virus detection with a rate of 1 positive in 3,624 collected midges (Vasconcelos et al. 1989).

Similarly, the knowledge of Oropouche virus reservoirs in Peru is limited. Some studies from the neighbor country of Brazil suggest that Oropouche virus reservoirs could include birds and several mammals species including sloths (*Bradypus tridactylus*), rodents (*Proechimys* spp.), and primates (*Callithrix* spp.) (Pinheiro et al. 1962, 1976, Nunes et al. 2005). Recently, active Oropouche virus surveillance in non-human primates in Brazil detected Oropouche virus in howler monkeys (i.e., *Alouatta caraya*) (Gibrail et al. 2016). We argue that similar studies should be conducted in Peru especially in the areas predicted of risk by our ecological models (Fig. 5).

Landscape perturbation has been proposed previously as a driver of Oropouche fever outbreaks. For example, in the 1962 outbreak in Bélem, Brazil, the construction of a highway was suggested as a causal factor for Oropouche fever emergence in the area (Pinheiro et al. 1962). Other studies have suggested landscape alterations as drivers of Oropouche fever transmission in the outbreak of San Martin department, Peru in 2010 (Alvarez-Falconi & Ruiz 2010) and Cutervo province, Peru in 2011 (Castro et al. 2013). Land cover information of the first record of Oropouche fever in Peru in Iquitos, 1992, or the recent Oropouche fever epidemic in Madre de Dios department, Peru, is lacking (García et al. 2016).

Spatiotemporal analysis of satellite imagery in this study suggests patterns of vegetation loss in the study area (Figs 3, 4). This land cover changes could help to explain Oropouche fever outbreak occurrence, although we are aware of our limitations in establishing causality. This exploration quantifies vegetation loss in sites with outbreaks supporting previous anecdotical proposals of deforestation as a factor facilitating Oropouche fever outbreaks. Thus, local studies at fine spatial resolution may help to identify associations between areas with Oropouche fever occurrence and vegetation loss compared to more ‘pristine areas.’ This initial assessment will help to elucidate magnitude and temporal lag of vegetation loss in generating reports of Oropouche fever transmission. The dilution effect could be one mechanism explaining why vegetation loss can be linked to Oropouche fever emergence. The dilution effect is an ecological theory suggesting that a reduction in the biodiversity could facilitate the increase on diseases; thus, according to the dilution effect a pathogen or parasite could increase in prevalence in a site if the overall richness of species decrease (Ostfeld & Keesing 2000).

Our areas identified as at risk for Oropouche fever transmission may be ideal candidates for monitoring land cover change, and also to guide active epidemiological surveillance for early detection of Oropouche fever cases. Interestingly, a retrospective serological survey in Madre de Dios department in December 2015 - January 2016 found up to 24% (122/508) seropositivity for Oropouche fever (García et al. 2016). Moreover, the World Health Organization (WHO) recently published an epidemiological survey in Madre de Dios during February 2016, finding mixed infections of dengue and Oropouche fever with at least 120 confirmed cases of the latter (WHO 2016). These results indicate ongoing circulation of Oropouche virus in regions geographically adjacent to the outbreak explored here, specifically in Lares (Fig. 1), and suggest that Oropouche fever could be misdiagnosed and confounded with dengue in view of the symptomatic similarity of both febrile diseases. The western part of Madre de Dios department, included in our study area, was also predicted as suitable for Oropouche fever by our model (Fig. 5). Thus, the recent reports of WHO may validate the predictions of our model in these areas and suggest that more areas could have ongoing sylvatic circulation of Oropouche virus in the sectors identified as highly suitable.

The lack of information regarding Oropouche-positive vectors and the limited information on reservoirs in Peru are other critical limitations for the understanding of Oropouche fever ecology. In consequence, we employed a black-box ecological niche modeling approach to characterise the distributional ecology of Oropouche fever at landscape level (Peterson 2014). A black-box approach considers human cases as the end point of the complex ecological interactions in the transmission dynamic of an infectious disease, identifying the joint intersection of all elements in the system (i.e., virus, reservoir, vector, host, landscape). As such, our results provide a guide for future fieldwork to test ecological and epidemiological hypotheses regarding Oropouche fever emergence in tropical Latin America. The maps deriving from our niche modeling efforts can help to prioritise economic and human resources for prevention efforts of Oropouche fever transmission in Cusco.
